# Development of image analysis tool to evaluate Langerhans cell migration after exposure to isothiazolinones

**DOI:** 10.1007/s00204-025-04013-3

**Published:** 2025-03-13

**Authors:** Christelle Oltramare, Olivier Burri, Nancy B. Hopf, Sandra Jaccoud, Arne Seitz, Lee Ann Applegate, Nathalie Hirt-Burri, Aurélie Berthet

**Affiliations:** 1 Department of Occupational and Environmental Health, Center for Primary Care and Public Health (Unisanté), University of Lausanne, 1066 Epalinges, Switzerland; 2 Swiss Center for Applied Human Toxicology (SCAHT), Basel, Switzerland; 3 Swiss School of Public Health (SSPH+), Zurich, Switzerland; 4 BioImaging and Optics Platform, Ecole Polytechnique Fédérale de Lausanne (EPFL), 1015 Lausanne, Switzerland; 5 Regenerative Therapy Unit, Lausanne University Hospital, University of Lausanne, 1066 Epalinges, Switzerland; 6 Center for Applied Biotechnology and Molecular Medicine, University of Zurich, 8057 Zurich, Switzerland; 7 Oxford OSCAR Suzhou Center, Oxford University, Suzhou, 215123 People’s Republic of China

**Keywords:** QuPath software, Skin sensitization, Allergic contact dermatitis, In vitro model, DAB-staining CD1a immunohistochemistry, Skin histology

## Abstract

Incidence of allergic contact dermatitis (ACD) is rising worldwide as a consequence of raising chemical exposure, especially from biocides present in common water-based products such as cosmetics and household products. Understanding the biological processes involved in skin sensitization is crucial to mitigating ACD. Migration of Langerhans cells to the basal lamina of the skin is a key step in sensitization. Animal testing for sensitization has been largely replaced by alternative in vitro methods due to ethical concerns. This study presents an open-source program using QuPath software enhanced with machine learning to analyze Langerhans cells migration in immunostained human skin samples. We validated scripts for automated detection of skin layers and Langerhans cells position, enabling accurate, reproducible analysis. An ex vivo human skin organo-culture model was used to test Langerhans cells migration after 24-h exposure to four different isothiazolinones diluted in water at different concentrations. The initial results show that the water vehicle has an impact on Langerhans cells migration. We also observed a different Langerhans cells migration pattern for methylisothiazolinone and benzothiazolinone exposed skin compared to octylisothiazolinone. The results suggest that only octylisothiazolinone can induce Langerhans cells migration to the basal lamina of the epidermis as described for sensitizers. This automated approach represents a advancement in ACD risk assessment by minimizing the subjectivity and labor involved in Langerhans cells analysis, providing a valuable tool for future research.

**Supplementary Information:**

The online version contains supplementary material available at 10.1007/s00204-025-04013-3.

## Introduction

Skin allergic contact dermatitis (ACD) represents an important global public health concern, largely attributable to the increasing exposure of the skin to a multitude of products, often water-based products used in everyday life (Teo et al. 2019; Uter et al. 2020; Faraz et al. 2024). Many of these products contain several ingredients, including chemicals that preserve the chemical composition. Many of these chemicals have been identified as skin sensitizers. Among the most prevalent are biocides, which are extensively used as preservatives in a variety of consumer products, including household products, cosmetics, and paints (Lidén et al. 2022). Two biocides are in particular known to cause allergic response: methylchloroisothiazolinone (MCI) and methylisothiazolinone (MI) (Boyapati et al. 2013; Castanedo-Tardana and Zug 2013; Capkin et al. 2017). Until the 2000s, MCI and MI were used as a 3:1 ratio mixture (with a maximum allowed dose of 15 ppm in the product) and MI was used alone at a maximal tolerated dose of 100 ppm (Rodrigues-Barata and Conde-Salazar 2013). After this change, there was a dramatic increase in the number of MI allergies reported in Europe (Lundov et al. 2011; Havmose et al. 2021). This led to the ban of MI as a preservative for leave-on cosmetic products and a restriction to 15 ppm in rinse-off cosmetics since 2017 (Table 1). This legal restriction results in a significant decrease in MI contact allergies in European countries. In contrast, the United States continues to experience an increase in the prevalence of ACD as MI is not strictly regulated (Reeder et al. 2023; Schwensen et al. 2024).

**Table 1:** Tested isothiazolinones and their maximal concentrations in cosmetics and as biocides in preservatives and disinfectants

Biocide	CAS no.	Tested concentrations	Use and maximal concentrations in cosmetics in European legislation	Other uses as biocides^a^	Maximum admitted concentration as biocide
Methylchloroisothiazolinone (MCI)	55,965-84-9	30 and 600 ppm	0.0015% (15 ppm, mixed with MI (3:1)^b^ in rinse-off (forbidden in leave-on)	(Mix MCI/MI 3:1) PT02/PT04/PT06/PT11/PT12/PT13	(Mix MCI/MI 3:1) paints, glues, detergents: < 15 ppm
Methylisothiazolinone (MI)	2682-20-4	30 and 600 ppm	0.01%^b^ (100 ppm)	PT06/PT11/PT12/PT13	Paints, glues, detergents: < 300 ppm
Benzothiazolinone (BIT)	2634-33-5	300 and 500 ppm	Not allowed^c,d^	PT02/PT06/PT09/PT10/PT11/PT12/PT13	Paints, glues, detergents: < 360 ppm
Octylisothiazolinone (OIT)	26,530-20-1	300 and 600 ppm	Not allowed^c,d^	PT06/PT07/PT08/PT09/PT10/PT11/PT13	Insufficient data

^a^*PT* product type; *Disinfectants–PT02* disinfectants and algaecides not intended for direct application to humans or animals, *PT04* food and feed area, *Preservatives–PT06* preservatives for products during storage, *PT07* film preservatives, *PT08* wood preservatives, *PT09* fibre, leather, rubber and polymerized material preservatives, *PT10* construction material preservatives, *PT11* preservatives for liquid-cooling and processing systems, *PT12* slimicides, *PT13* working or cutting fluid preservatives (EUR-Lex 2012)

^b^EUR-Lex 2023

^c^Friis et al. 2014

^d^Allowed in cosmetics in the US and Canada (Silva et al. 2020)

Isothiazolinones have a potent antimicrobial activity at very low concentrations due to their electrophilic nitrogen–sulfur (N–S) bonds, which react with nucleophilic cell material and oxidizes compounds containing thiols. Although some isothiazolinones, such as benzothiazolinone (BIT) and octylisothiazolinone (OIT), are restricted in cosmetics, they are still used in many household products (Silva et al. 2020). Current research on skin sensitization to biocides primarily focuses on cosmetic products, leaving a knowledge gap in understanding ACD induced by other domestic products.

Reducing the occurrence of ACD is necessary and the first step is to understand the biological processes of skin sensitization induction after exposures to chemicals. Skin sensitization is an immunological response that occurs when a substance triggers an allergic response following skin contact (Robinson et al. 2000; Nestle et al. 2009). The biological key events are well reported and an adverse outcome pathways scheme has been developed (Sakuratani et al. 2018; de Souza et al. 2024). Sensitization consists of two phases, where first a substance induces a response when it penetrates the epidermis and constitutes the direct molecular initiating event (Ryan et al. 2001). Primary skin cells (keratinocytes) become activated (key event (KE) 1; KE1) and release proinflammatory cytokines and chemokines (KE2). Langerhans cells are then activated and migrate to lymph nodes in order to present the allergen to T lymphocytes (KE3) (Robinson et al. 2000; Wong et al. 2015). Langerhans cells are located in the basal and suprabasal layers of the epidermis and are a type of dendritic cell representing highly specialized antigen-presenting cells. This first phase is clinically silent while the second phase, the inflammatory elicitation that occurs after a re-exposure to the same substance, is visible and manifested as ACD. This is the adverse outcome in the adverse outcome pathways scheme (Basketter and Maxwell 2007).

Skin sensitizers were previously identified and assessed for sensitizing properties using animal testing. European prohibition of animal use in cosmetics, however, requires alternative models. Currently, the Organization for Economic Co-operation and Development (OECD) program has validated three in vitro methods as test guidelines (Bergal et al. 2020). Migration of the Langerhans cells to the lymph nodes to present the allergen to T lymphocytes (KE3) has been assessed in several studies mainly using a human monocytic leukemia cell line, in the human Cell Line Activation Test (h-CLAT). This test quantifies changes in the expression of cell surface markers related to dendritic cell activation. Nevertheless, few studies have evaluated the necessary activation of Langerhans cells by measuring their function and downward migration in the epidermis (KE2-KE3) (Pistoor et al. 1996; Rustemeyer et al. 2003; Lehé et al. 2003, 2006; Ouwehand et al. 2008, 2010; Rodrigues Neves and Gibbs 2021).

Human skin explant cultures have been shown to be a suitable model to assess contact allergen effect on Langerhans cells migration (Pistoor et al. 1996; Ryan et al. 2001; Rustemeyer et al. 2003; Lehé et al. 2003, 2006; Ouwehand et al. 2008, 2010, 2011). These studies have shown an accumulation of the Langerhans cells at the epidermal-dermal junction after a 24-h exposure to allergens. Most of the studies assessing Langerhans cells migration used manual counting of Langerhans cells visible on the immunostained slides (Pistoor et al. 1996; Rustemeyer et al. 2003; Lehé et al. 2003, 2006). This process is time consuming and needs several observers to avoid subjectivity. In 2001, Jacobs et al. published an automated Langerhans cells cell counting program based on the Leica QWin program, a software linked to a microscope. This expensive program estimates the number of CD1a-stained Langerhans cells. For 20 years, the artificial intelligence improved considerably the automatic reading of imagining. With Open-source program, it is now possible to detect cells as well as different tissues.

In this study, we provide an open-source bioimage analysis program (QuPath) for detecting and counting DAB-stained CD1a Langerhans cells distribution throughout the epidermis in histology slides (irrespective of microscope type). The bioimage analysis program has a first script that automates the detection of the epidermis and the dermis on immunohistological images through machine learning, and a second script that quantify the Langerhans cells distribution in the epidermis. It includes fully documented and open image analysis features such as machine learning pixel classifiers, which we used to distinguish the different skin layers in DAB-stained skin cross-sections. We applied the newly developed bioimage analysis program on immunohistological images obtained from our human skin experiments with four isothiazolinones: methylchloroisothiazolinone (MCI), methylisothiazolinone (MI), benzothiazolinone (BIT), and octylisothiazolinone (OIT).

## Materials and methods

### Isothiazolinone

These four isothiazolinones were selected because of their common use and potential of inducing skin sensitization (Friis et al. 2014). The concentrations of isothiazolinones tested on the skin were determined based on the regulatory limits established for biocide products, including cosmetic products containing MCI and MI in Europe, and BIT and OIT in the United States (see Table 1). Isothiazolinone standards in powder formulation were purchased from Dr. Ehrenstorfer (Germany), for MCI with a purity of 97% and from Sigma-Aldrich (United States) for MI (ref 725,765), BIT (ref 561–487) and OIT (ref 46,078).

### Skin samples

#### Ethical compliance

Skin samples of full-thickness abdominal skin were obtained from ten anonymous donors from the Department of Musculoskeletal Medicine at the CHUV (Lausanne University Hospital, Lausanne, Switzerland (DAL) biobank registered in the CHUV-DAL Department Biobank according to an approved ethical committee protocol and biobanking directive (BB_029_DAL).

#### Experimental design

**Fig. 1:**
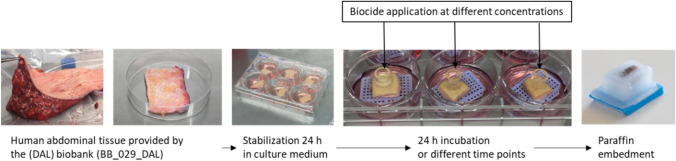
The standard operating procedure (SOP) for the skin exposure experiments

The full-thickness abdominal skin was delivered approximately 2–6 h after the start of the surgery as surgical waste from anonymous donors. The fat was removed from the skin under a sterile laminar hood to a thickness of approximately 2–4 mm. After cleaning the skin with phosphate-buffered saline 1 × solution (PBS, Bichsel, Unterseen, Switzerland) containing 1% antibiotics (penicillin/streptomycin), the skin was cut into 12 skin samples with a scalpel into squares of approximately 1 cm^2^ and placed in a 12-well culture plate. The skin samples were stabilized for 24 h at 37 °C, 95% humidity, and 5% CO_2_ in a cell culture incubator using a cell culture medium containing DMEM (Thermo Fisher Scientific, Waltham, MA, USA) and Ham’s F12 (Merck, Darmstadt, Germany) in a 3:1 proportion, with 20 µg/mL gentamycin, 0.14 nM cholera toxin (Lubio Science, Zurich, Switzerland), 400 ng/mL hydrocortisone (Pfizer, New York, NY, USA), 8.3 ng/mL EGF (Merck, Darmstadt, Germany), 832.2 µM L-glutamine, 0.12 U/mL insulin (Novo Nordisk Pharma, Bagsværd, Denmark), and 10% FBS (Merck, Darmstadt, Germany). Following this stabilization step, each skin sample was placed on an air–liquid bridge in a 6-well container, as shown in Fig. 1. A step-by-step procedure is detailed in the supplementary Table S1.

The skin surface was gently dried with a sterile cotton swab, and an 8 mm diameter glass insert was placed on the top of the skin and filled with 20 μL of aqueous isothiazolinone. After 24 h of exposure, the insert was removed, and the exposed part of the skin was sampled using a round cutter of 8 mm. Samples were cut in half, fixed in formalin (Sigma Aldrich, Buchs, Switzerland) for 24 h at room temperature, rinsed with PBS, and placed in 70% ethanol at 4 °C until fixed in paraffin. The tissue blocks included in paraffin wax were sectioned to a thickness of 7 μm using a microtome (LEICA RM2235) and placed on a sperfrost™ plus glass slide (Epredia, United States). The slides were kept at room temperature until the immunohistochemistry was performed and Fig. 1 illustrates the entire procedure and summarized in Table S1).

The isothiazolinones were diluted in sterile water at different concentrations as reported in Table 2 according to regulation cutoffs and tested separately giving in total 10 experiments (Table 3). One experiment corresponds to one donor. Depending on the size of the sample for each donor, we had between 7 and 26 sections of 1 cm^2^. Each experiment was performed in triplicate, and each concentration was also assessed with a minimum of three replicates unless otherwise noted. Controls were taken at 0 (T0) and 24 (T24) h for both untreated skin and skin treated with the vehicle (sterile water), as shown in Table 3.

**Table 2:** Skin exposure concentrations based on the addition of 20 µL of the isothiazolinone solution within an 8 mm diameter insert

	Mass molar	Concentration in solution			Mass applied on skin	Concentration in 20 µL
	(g/mol)	(ppm)	(g/L)	(%)	(µg)	(g/m^2^)
MCI	149.60	30	0.03	0.003	0.6	0.012
MI	115.15	30	0.03	0.003	0.6	0.012
MI	115.15	300	0.3	0.03	6	0.12
BIT	151.19	300	0.3	0.03	6	0.12
BIT	151.19	500	0.5	0.05	10	0.19
OIT	213.34	300	0.3	0.03	6	0.12
OIT	213.34	600	0.6	0.06	12	0.23

**Table 3:** Summary of the skin experiments including number of replicates for each condition

Skin sample		Age of the donor (years)	Control samples			Isothiazolinone-exposed samples						
No.	Name		Ctrl T0	Ctrl T24	H_2_O T24	MCI	MI		BIT		OIT	
						30 ppm	30 ppm	600 ppm	300 ppm	500 ppm	300 ppm	600 ppm
1	SSM_exp1	59	2	3	3	nt	nt	nt	nt	nt	nt	Nt
2	SSM_exp2	38	4	5	5	nt	nt	nt	nt	nt	nt	Nt
3	SSM_exp3	68	3	4	5	nt	nt	nt	nt	nt	nt	Nt
4	SSM_exp4	32	3	4	4	5	5	5	nt	nt	nt	Nt
5	SSM_exp5	75	3	3	5	5	5	5	nt	nt	nt	Nt
6	SSM_exp6	51	4	4	4	4	4	4	nt	nt	nt	Nt
7	SSM_exp7	28	3	3	5	5	5	5	nt	nt	nt	Nt
8	SSM_exp8	38	2	2	4	nt	nt	nt	4	4	4	4
9	SSM_exp9	45	2	2	3	nt	nt	nt	nt	4	nt	4
10	SSM_exp10	47	2	2	3	nt	nt	nt	4	4	4	4

Each experimental skin sample comes from a single donor. Exposed samples were compared to control samples, designated as either “Ctrl” (only viable skin) or “H_2_O” (only vehicle without isothiazolinone). The control viable skin samples were sampled at the beginning of the experiment (T0) and after 24 h of exposure (T24)

*nt* not tested

### Immunohistochemistry

The paraffin-included tissue sections were deparaffinized by immersion in xylene twice for 10 min each, followed by two rounds of 100% ethanol for 10 min each, one round of 90% ethanol for 10 min, and one round of 74% ethanol for 10 min. Incubating the sections with H_2_O_2_ (Hydrogen peroxide solution, 30% Sigma-Aldrich) (10% in 1 × PBS) at 60 °C for 1 h blocked the endogenous peroxidase activity and bleached the melanin. The sections were then washed at room temperature for 10 min (0.1%Triton Tx-100, Triton (TM) X-100, X100-5ML, Merck) and incubated with 2.5% horse serum (MP-7402–50 ImmPRESS™ anti-Mouse IgG HRP Polymer Detection Kit, made in Horse, VectorLabs) for 1 h. The slides were incubated overnight at 4 °C with a mouse anti-CD1a antibody (1/1000, Thermofisher, MAS—12,226) that specifically recognizes human Langerhans cells. The next day, after washing the slides, the appropriate secondary antibody (MP-7402-50 ImmPRESS™ anti-Mouse IgG HRP Polymer Detection Kit, made in Horse, VectorLabs) was added and incubated for 1 h. The activation was performed with ImmPact DAB (Peroxidase revelation with ImmPACT® DAB Substrate Kit, Peroxidase (HRP) (SK-4105), VectorLabs). After immunohistochemical staining, the slides were counterstained with fastRed (Nuclear fast red solution, #N3020-100 Fluka) and mounted on resin and glass coverslips (Mounting medium EUKITT, vol. 100 ml). A negative control was obtained by omitting the primary antibodies. Each slide consisted of two sections, positive CD1a-stained Langerhans cells and a negative control.

### Image acquisition

The entire surfaces of the slides were scanned using an Olympus FL-120 microscope with a UPLSAPO 20x/0.75 objective and a Pike F505 Color camera. Acquisition and stitching were performed during acquisition using the defaults from the Olympus VS ASW version 2.9 software. The resulting images (.vsi format) were imported into QuPath for processing and analysis.

### Image processing and analysis

**Fig. 2:**
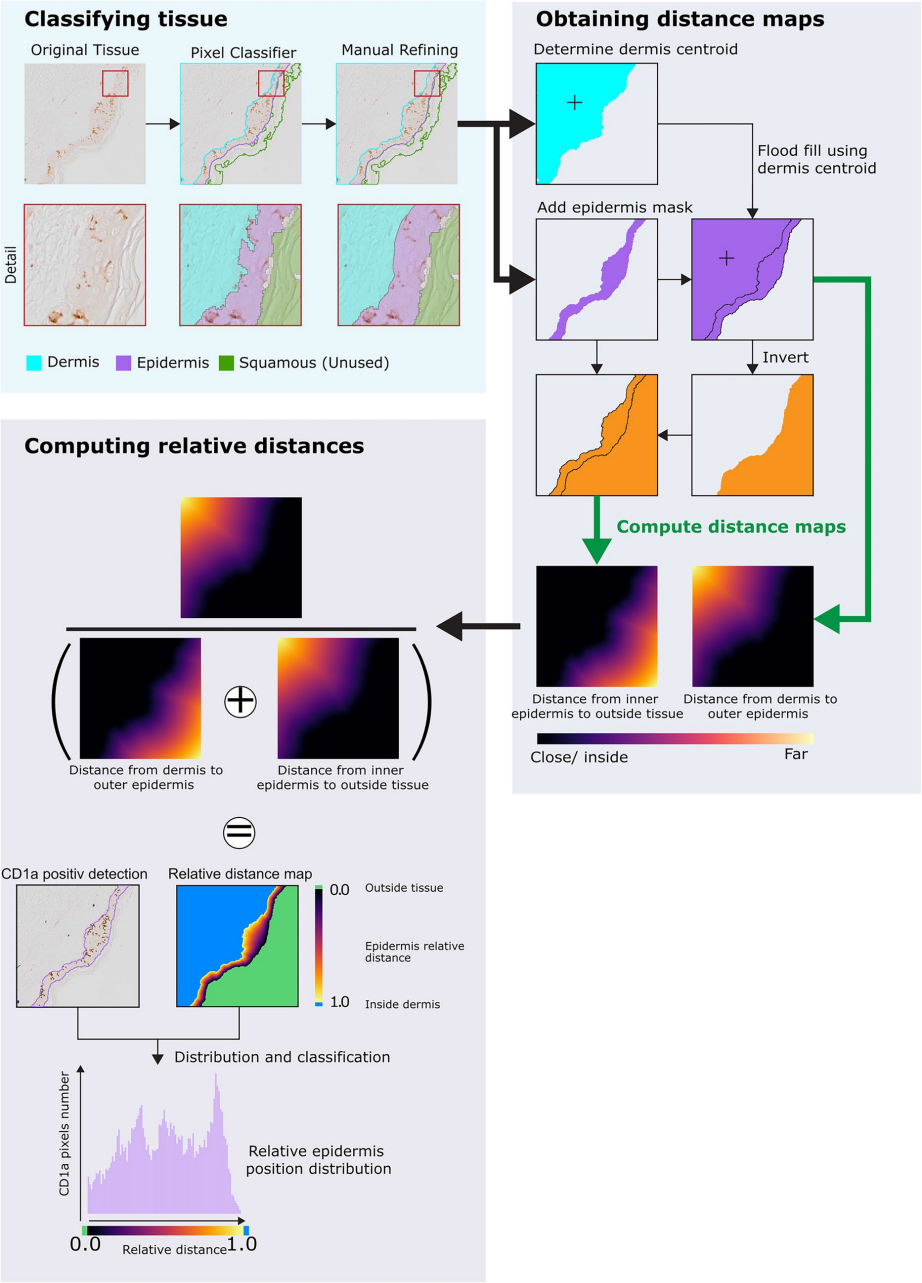
QuPath workflow: annotations for dermis, epidermis and squamous layer were obtained from a pretrained pixel classifier. The borders were manually refined to better reflect the dermis/epidermis interface. From these, we extracted and computed binary masks in order to obtain distance maps of inner epidermis to outer tissue and dermis to outer epidermis. The distance maps allowed to compute a relative distance metric for all pixels in the epidermis. Finally, DAB-positive signal was segmented using QuPath’s cell detection (Table 4). The average distance and distance distributions were measured within the detected DAB areas for further analysis

The acquired images were processed using QuPath 0.3.0 or 0.4.3. A schematic workflow is illustrated in Fig. 2. Briefly, a single Pixel classifier was obtained and optimized for all experiments, as staining showed minimal variability across batches and conditions. We designed the Pixel classifier to recognize three different tissue features (classifications): squamous, epidermis and dermis. The three different tissue classifications were manually annotated on subsamples across multiple images to train the object classifier. The trained classifier was then applied to 3–5 squares regions on the whole slide image to cover different part of the sample and ensure an adequate representation of the sample. These regions were manually defined by an operator, to avoid parts of the tissue that may present artifacts, such as folds or cuts.

Two scripts have been developed and are available online (https://github.com/BIOP/qupath-skin-migration). The first script runs the pixel classifier on the operator annotated regions. At this point, the operator has the possibility to manually inspect and refine the boundaries of each classified area using QuPath’s annotation-edition tools, namely brush tool (Fig. 2: pixel classifier—> manual refining). The second script detects the areas exhibiting DAB staining, representing presence of CD1a in each square region and computes the relative distance of these areas to the dermis–epidermis interface (Fig. 2). To obtain the relative distance, two binary masks are created, based on the results of the pixel classifier and user refinement. The first contains the epidermis and dermis regions. It is achieved by creating a binary image of the epidermis region and performing a flood fill at the centroid position of the dermis mask. Since the subsequent distance map calculation is extremely sensitive to the presence of even a single non-dermis pixel in the dermis area, simply using the dermis area directly from the pixel classifier could cause errors in the distance map. This approach was effective because the epidermis extends across the entire square region, creating a partition of the where the dermis and the outside would never be in contact. The second mask consists of the outside of the sample and the epidermis. This mask is created by inverting the epidermis + dermis mask and re-adding the epidermis region. Euclidean distance maps (EDM) that represented (i) the distance from the top of epidermis (*D*_top epidermis_) and (ii) the distance from the top of the dermis (*D*_top dermis_) can then be directly calculated. We used the following equation (Eq. 1) to obtain a relative distance (*d*_relative_) from the top of the epidermis:

1 $$ d_{{{\text{relative}}}} = \, {{D_{{\text{top epidermis}}} } \mathord{\left/ {\vphantom {{D_{{\text{top epidermis}}} } {\left( {D_{{\text{top epidermis}}} + \, D_{{\text{top dermis}}} } \right)}}} \right. \kern-0pt} {\left( {D_{{\text{top epidermis}}} + \, D_{{\text{top dermis}}} } \right)}} $$

The automatic DAB area detection was performed using QuPath’s Built-in “Watershed cell detection” algorithm. The parameters used are summarized in Table 4. In summary, the algorithm applies a series of preprocessing steps to ensure robustness to biological variability in local intensity changes, followed by an intensity threshold based on DAB signal intensity, ending in a connected components analysis to cluster touching areas, and finally a size filter. The result is a series of distinct areas that are positive for CD1a. Because of the complex morphology of LCs, and the 2D nature of the tissue sections, actual cell counts are meaningless. We thus used the detected areas. We first compute the relative distance of each detected blob. The script calculates the relative distance for each CD1a blob using Eq. 1. A value of 0 is assigned to areas pixels outside the sample (epidermis or dermis), while a value of 1 was assigned to areas in the dermis. For areas in the epidermis, their relative distances to the epidermis edges were represented by a value of 0.0 if they were at the interface between the squamous and the epidermis, and 1.0 if they were at the interface between the epidermis and the dermis. This allows to calculate the average distance from the epidermis and the average distance from the dermis for area in µm, in addition to the relative distance. To prevent bias based on “blob” area (where large areas would have the same weight as small areas), the script also returns a histogram of the absolute and relative distance for each CD1a positive pixel, which can be visualized as histograms or violin plots.

**Table 4:** Optimized parameters for the automated cell detection in QuPath

Parameter	Value
Detection channel	Optical density sum
Pixel size	0.6892 µm
Background radius	20 µm
Median radius	0 µm
Gaussian blur sigma	2 µm
Minimum area	5 µm
Maximum area	40,000 µm
Threshold	0.20
Maximum background intensity	2.0
Split by shape	False
Exclude DAB	False
Cell expansion	0 µm
Include nuclei	True
Smooth boundaries	True
Make measurements	True

### Optimization of the standard operating procedure and the QuPath model

Several parameters were optimized regarding the standard operating procedure. At the experimental level, the stabilization and exposure duration were evaluated. The material for the chemical exposure (glass insert or blotting paper) was also part of the optimization. Then, regarding the QuPath procedure, we assess mainly the surface of the image analyzed.

### Statistical analyses

The normality of each condition for each skin sample was tested using a Kolmogorov–Smirnov test. As the p value was lower than 0.05, the data were not normally distributed. To compare the distribution of the control skin samples with the exposed skin samples, we used the non-parametric Wilcoxon test. The null hypothesis (*H*_o_) was that Y1 and Y0 have a similar distribution. In addition, we tested the null hypothesis (*H*_o_) that Y1 is greater than Y0. The alternative hypothesis was that there is a significant difference. Statistical significance was defined as *p* value < 0.05. The Wilcoxon test was performed on both the total distribution and the distribution divided in three equal parts. The statistical analysis was performed in R with Rstudio (version 2023.03.0).

The results are presented using violin plots to visualize the distribution of pixel counts for positive DAB-staining. Each violin plot represents the mean of sample replicates for each skin sample (equivalent to each donor). The figures show the relative distribution of the pixels position in the epidermis. These figures show normalized value (area under the curve is normalized). We are more interested in the shape rather than the number of pixels counted.

## Results and discussion

### Model validation

#### Optimization of the standard operating procedure and the data acquisition

The data acquisition process in QuPath was optimized by automating the steps and thus save time during image processing. The main optimization focused on the area of the slide that was analyzed, which could be either the entire image or several squares selected by the operator. Typically, the entire image contained two scanned skin slides. We did not observe any significant differences between the two methods (data not shown). The ‘square’ method was preferred because the method of analyzing the entire image required more time to run the program and additional investment to correct areas manually by the operator.

#### Evaluation of the parameters to assess the skin baseline of Langerhans cells distribution

**Fig. 3:**
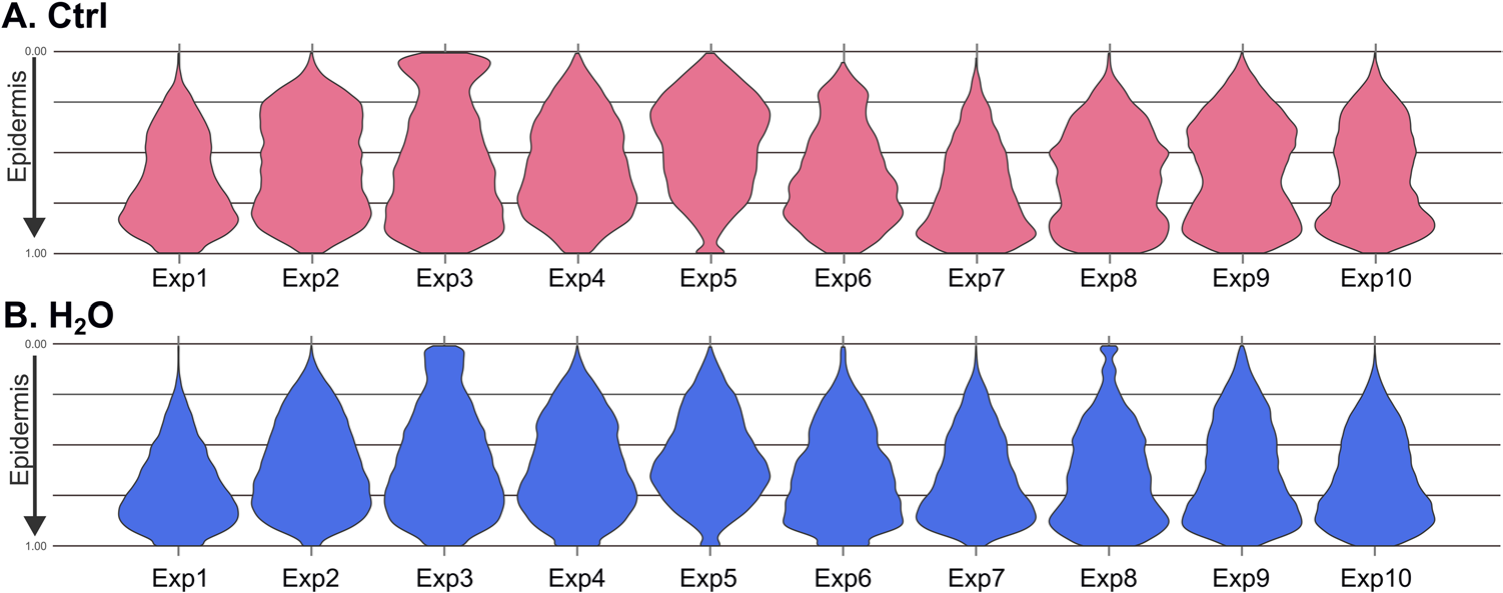
Distribution of DAB-stained pixel count in the epidermis after 24 h in the control and H_2_O exposed of each skin samples (*n* = 10). **A** Ctrl—control unexposed skin. **B** H_2_O (vehicle) exposed skin. Each violin plot represents the mean of sample replicates for each skin sample. Between 3 and 5 section were used for each experiment (Exp). In the *Y* axis, the 0.00 point represents the stratum corneum and the 1.00 point represents the position of the basal lamina. The *p* value is obtained from the Wilcox test between the two samples conditions (Ctrl: no exposure, H_2_O: only the vehicle). With a *p* value < 0.05, showing a significant difference between the compared samples conditions

There is currently no research on the distribution of Langerhans cells when the skin is not exposed to sensitizers. Jacobs et al. (2001) have reported the total number of cells in their paper with no mention of their position within the epidermis. The initial step was to examine the distribution of DAB-stained pixel between the day of surgery (*T*_-1_, *n* = 7, 2–4 replicates), the beginning of exposure to isothiazolinones (after 24 h of stabilization, referred to as *T*_0_, *n* = 10, 2–4 replicates), and the end of the 24-h exposure to chemicals (*T*_24_, *n* = 10, 2–5 replicates). The distribution of DAB-stained pixel on the day of surgery (*T*_-1_) was too unstable between the replicates to establish a baseline (Figure S1 in supplementary data). The distribution of DAB-stained pixel at the end of the experiment (*T*_24_) was found to be the most reproducible across the experiments, although, there was a large variation across individuals, as illustrated in Fig. 3A. As expected, due to the high inter-individual variation, it was not possible to assess a standardized baseline across the different experiments. Our observations show that the tendency of DAB-stained pixel referring to Langerhans cells, distribution through the epidermis is mainly in the middle with a double peak rather than a Gaussian distribution. From now on, we will refer to T_24_, unexposed skin taken at the end of the experiment as the control (Ctrl).

#### Effect of H_2_O exposure on Langerhans cells distribution

We have found that water exposure for a period of 24 h in our model setting resulted in a shift of the distribution of the DAB-stained pixel across the epidermis as represented in Fig. 3B. Violin plots look like conifer trees the more Langerhans cells had migrated from the stratum corneum to the basal lamina. The distribution shift was observable for almost all samples. The non-parametric Wilcoxon test confirmed that the DAB-stained pixel distribution across the epidermis was statistically different for 9 of the 10 experiments (Fig. 3B). This suggested that exposure to water may induce an allergic reaction by Langerhans cells migration. In 1992, Mikulowska (Mikulowska 1992) described this phenomenon and demonstrated that Langerhans cells was able to migrate when the skin was subjected to water occlusion. The application of substances in aqueous solutions on the skin may induce an additive effect when combined with occlusion. This is further supported by studies reporting that water can impact the mechanical properties of the stratum corneum and the structure of full-thickness skin (Dhandapani et al. 2020). On the other hand, Willis (1973) noted that inflammation was induced in the skin after exposure to water for 72–144 h. Overall, our results confirmed that water exposure affected the Langerhans cells distribution in the epidermis, suggesting a potential mechanism for water-induced allergic reactions. This highlighted the necessity of considering water exposure in skin sensitization experiments.

Laboratory procedures often involve testing reactions using a patch, typically on blotting paper (Robinson et al. 2000; Basketter 2009). In our experiments (Exp1; Exp2; Exp3), we validated the use of the insert glassware, as an exposure reservoir, in comparison with the blotting paper method. We observed no significant differences between the two methods (data not shown) for the DAB-stained pixel distribution across the epidermis when skin was exposed to water. This demonstrated the reliability of our experimental approach.

### Test of the model with isothiazolinones

The regulations for cosmetic products differentiate the maximum allowable concentration depending on whether products are classified as “leave-on” or “rinse-off” products. While OIT and BIT are prohibited in cosmetics in the EU, they are permitted in the US. In Europe, these substances may be used as biocides in other products, including domestic and industrial products (see Table 1) (Silva et al. 2020). The maximal concentration permitted for these two products in other biocide applications is not well documented. Their presence were quantified in diverse products available in the Swiss market, with concentrations ranging from 5 to 279 ppm for BIT and 7.9 ppm for OIT in 2015 (Garcia-Hidalgo et al. 2017b, a).

#### Methylchloroisothiazolinone (MCI) and methylisothiazolinone (MI)

**Fig. 4:**
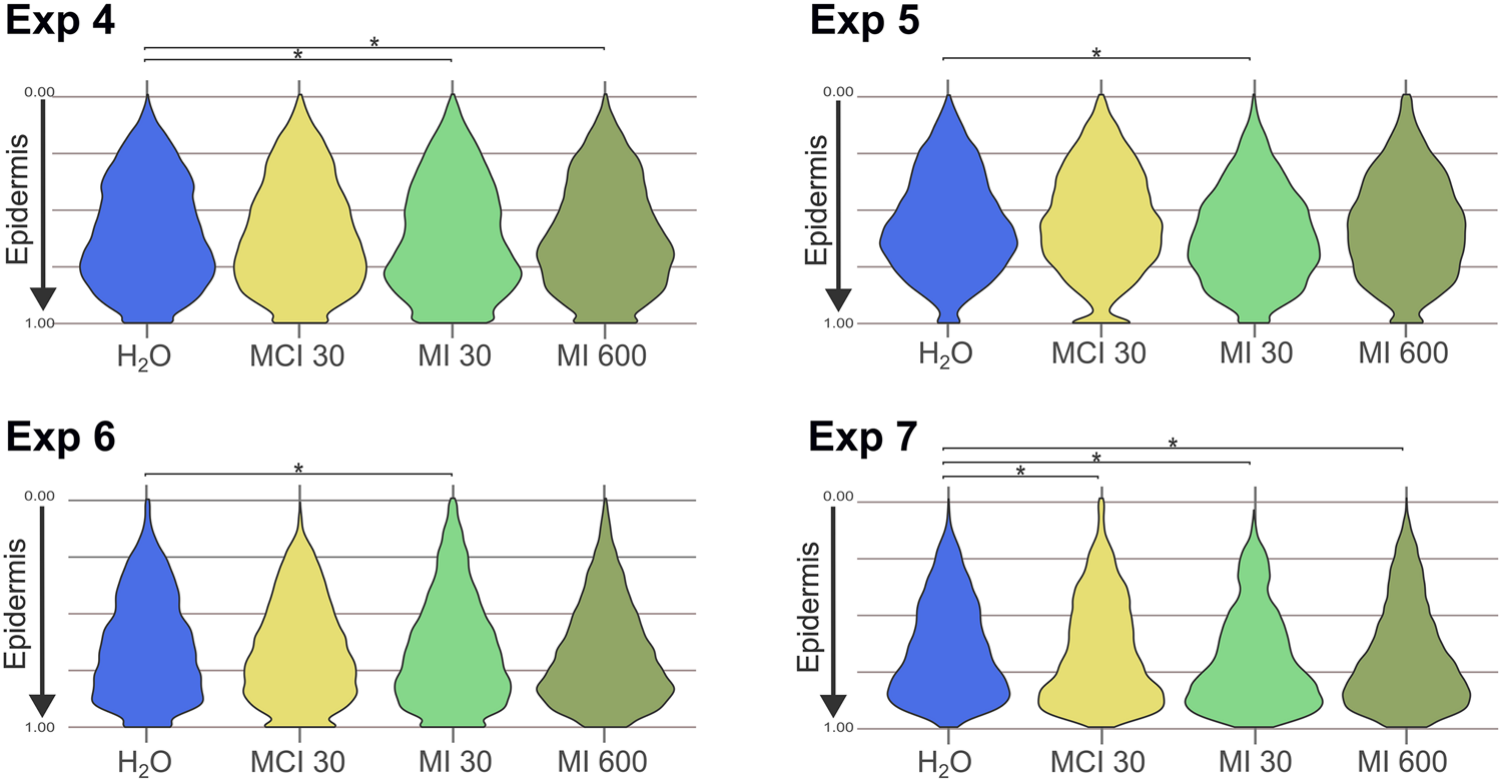
Distribution of DAB-stained pixel counts in the epidermis for the vehicle (H_2_O) and the exposure to MCI (30 ppm) and MI (30 and 600 ppm) in 4 independent experiments corresponding to 4 skin samples. Between 3 and 5 sections were used for each experiment (Exp). On the *Y* axis, the 0.00 point represents the stratum corneum and the 1.00 point represents the position of the basal lamina. (Significant code: *p < 0.01)

Figure 4 shows four experiments with water and MCI or MI in different concentrations (MCI 30, MI 30 and MI 600) and different donors (Exp 4–7) in replicates that are compared to water. Visually, we observe that the conifer tree shapes (of the violin plots) have a broader base for the MI condition. We observed a significant statistical difference in the DAB-stained pixel distribution across the epidermis between the 30 ppm MI condition and the vehicle (H_2_O) condition for the four tested skin sample (Fig. 4), indicating that exposure to MI induced a skin sensitization reaction by inducing Langerhans cells migration through the epidermis. In contrast, no statistical difference in DAB-stained pixel distribution was observed in 3 out of 4 experiments when skin was exposed to 30 ppm MCI. This may be because the dose tested for MCI was not sufficient to induce Langerhans cells migration across the epidermis, suggesting that the maximum admitted concentration established by regulation is conservative and appropriate for cosmetic and other biocidal uses.

Another potential explanation for MCI results could be that the concentration tested was closer to a threshold irritant dose. Previous studies demonstrated that skin irritation can result in direct Langerhans cells migration to the dermis and a reduction in the number of Langerhans cells in the epidermis (Kimber et al. 2009). While the evaluation of Langerhans cells numbers was not feasible using our program, a visual analysis of CD1a immunohistology slides for MCI suggested a reduction in the number of DAB-stained pixel in the epidermis compared to the dermis (Fig. 4). The capacity of Langerhans cells to migrate to the dermis following exposure to irritants could be due to the death of keratinocytes (Jacobs et al. 2004). Keratinocytes stimulate Langerhans cells migration in a non-antigen-dependent manner. A minimum of 8 h was sufficient for Langerhans cells to migrate out of the epidermis prior to cell death due to toxicity (Jacobs et al. 2006). In a previous study, we evaluated the ability of MCI and MI to permeate through the skin at different concentrations (ranging from 1 to 743 ppm for MCI and from 1 to 175 ppm for MI). MCI permeated the skin only at the highest concentration with a lag time of 3.5 h. In contrast, MI permeated the skin readily after 15 min at the lowest tested concentrations (1 and 50 ppm) (Berthet et al. 2013). This provides further evidence that chemicals classified as strong skin sensitizers in human patch tests show stronger irritancy potential than those classified as weaker skin sensitizers.

Similarly, at a dose of 600 ppm of MI, the results showed that the DAB-stained pixel distribution differed significantly from the control exposed to H_2_O (vehicle) in 2 out of 4 tested skin samples and from the MI concentration of 30 ppm. Furthermore, as observed for MCI, the visual analysis of CD1a immunohistology slides for MI suggests a higher number of Langerhans cells in the dermis compared to the epidermis (Fig. 4). This result tends to support the hypothesis that at 600 ppm, MI probably acts more as a skin irritant rather than a skin sensitizer, whereas at 30 ppm, MI is a skin sensitizer. Our results suggest that MI may induce sensitization at the maximum admitted concentrations for cosmetic use (100 ppm) and for paints, glues, and detergents (300 ppm). However, we could not assess the potential irritant effects of concentrations below 600 ppm, as they were not tested.

#### Benzothiazolinone (BIT) and octylisothiazolinone (OIT)

**Fig. 5:**
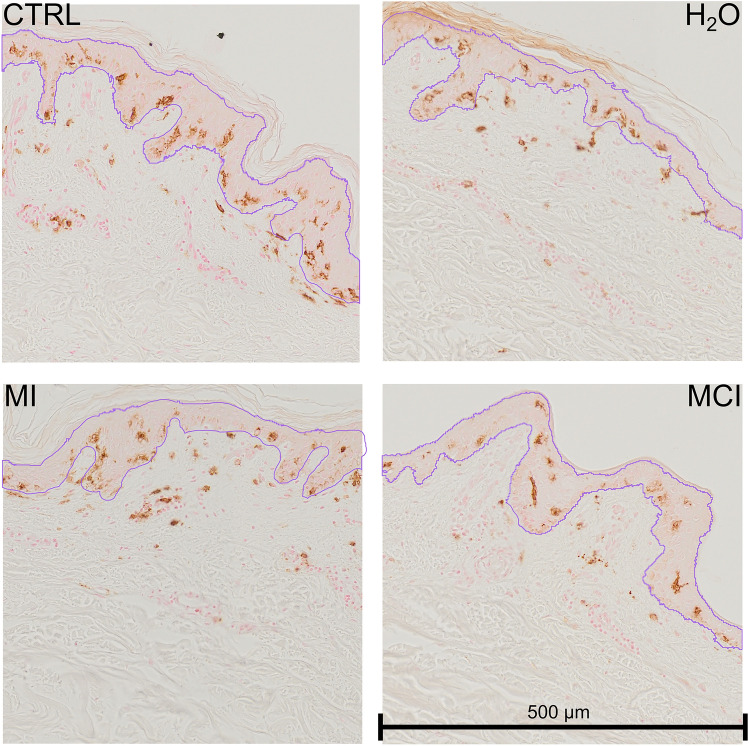
Distribution of DAB-stained pixel counts in the epidermis for controls exposed to H_2_O (vehicle) and for skin exposed to BIT (300 and 500 ppm) and OIT (300 and 600 ppm) in 3 independent experiments. Between 3 and 5 sections were used for each experiment (EXP). On the *Y*-axis, the 0.00 point represents the stratum corneum and the 1.00 point represents the position of the basal lamina (significant code: *p < 0.01)

There are very few publications on BIT and almost none on the sensitization potential of OIT. These biocides are prohibited in cosmetic products; however, they are authorized for use in other products, including paintings, adhesives, and detergents (Table 1). The maximum concentration of BIT permitted is 360 ppm, but none is available for OIT. To the best of our knowledge, it is the first time that OIT is tested on an organo-culture skin model. The skin was exposed to 300 and 500 ppm of BIT and 300 and 600 ppm of OIT for 24 h. Figure 5 illustrates the changes in DAB-stained pixel distribution in the epidermis. Visually, we can observe differences in the conifer tree shapes, specially between H_2_O and OIT. The base is broader for the OIT condition, indicating Langerhans cells had migrated from the stratum corneum closer to the basal lamina. The distribution of DAB-stained pixel is statistically different between the two OIT concentrations and the control exposed to H_2_O (vehicle), suggesting a sensitization reaction at the two tested concentrations. The plot patterns for the tested concentrations of OIT (300 and 600 ppm) applied on skin completely differed from patterns observed for MCI and MI. In contrast, the results for BIT showed a DAB-stained pixel distribution pattern comparable to that observed in MCI and MI exposure experiments (Fig. 4 and Fig. 5), with a significant difference in DAB-stained pixel distribution compared to the control exposed to H_2_O (vehicle) in 2 of the 3 experiments. Despite variability in results, BIT may be considered as a potential skin sensitizer at the two tested concentrations.

**Fig. 6:**
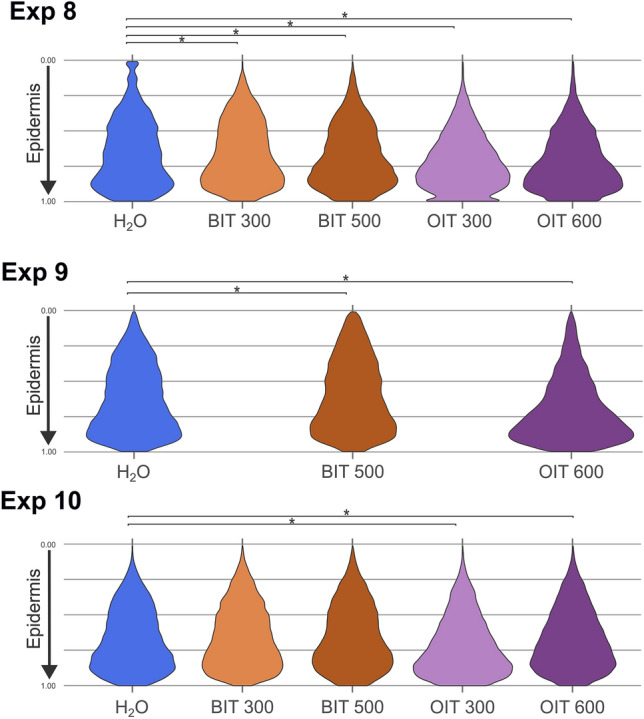
CD1a immunohistochemistry staining from experiment Exp9 for the following four conditions: control unexposed skin (Ctrl), exposure to water, exposure to BIT 500 ppm, and exposure to OIT 600 ppm. Each square is 500 × 500 µm size. The purple areas represented the epidermis computed from the script after manual refining by the operator (Fig. 2)

**Fig. 7:**
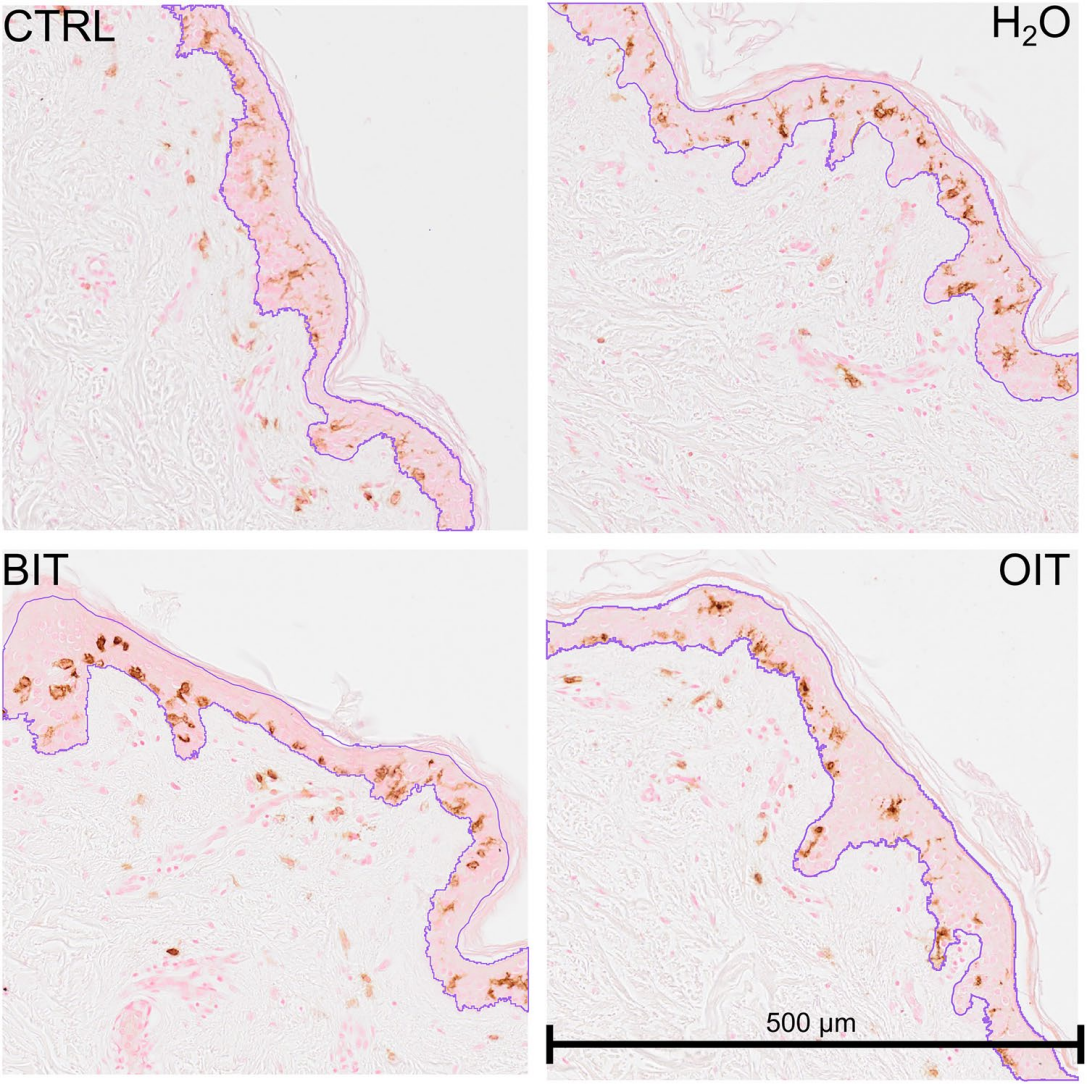
CD1a immunohistochemistry staining from experiment Exp 6 for the following four conditions: control unexposed skin (Ctrl), exposure to water, exposure to MI 30 ppm, and exposure to MCI 30 ppm. Each square is 500 × 500 µm in size. The purple areas represented the epidermis computed from the script after manual refining by the operator (Fig. 2)

Upon analysis of the CD1a immunohistochemistry illustrative slides (Fig. 6), we observed that DAB-stained pixel appeared to be located at the interface of the basal lamina in the epidermis for OIT, with limited DAB-stained pixel observed in the dermis. In contrast, when the skin was exposed to BIT, a greater number of DAB-stained pixel appeared to be in the dermis rather than in the epidermis, as also observed for MI at 600 ppm and MCI exposure (Fig. 7). Langerhans cells could be observed in the dermis as they migrated to the draining lymph nodes, where they presented antigen to T cells, thereby initiating then an immune response (Fukunaga et al. 2008; Clayton et al. 2017). Nevertheless, further studies are required to confirm Langerhans cells distribution differences between the epidermis and the dermis following exposure to MI, BIT, and OIT, testing for similar concentration. In addition, it would be valuable to investigate the gene expression related to irritation and sensitization in keratinocytes exposed to these isothiazolinones.

Overall, the results highlight a distinct sensitization potential of OIT compared to BIT, MI, and MCI. Skin exposure to OIT showed a statistically significant difference in DAB-stained pixel distribution between the different concentrations and control, suggesting a clear sensitizing response, at least at 300 ppm. Due to the limited data available on OIT, further studies are necessary to define a maximum concentration that does not induce skin sensitization. Skin exposure to BIT shows a variability in the DAB-stained pixel distribution pattern, indicating the potential for a sensitizing response. However, its DAB-stained pixel distribution pattern closely aligns with that of MI at 600 ppm and MCI, suggesting the possibility of skin irritation at the tested concentrations. In light of the current results suggesting potential skin sensitizing at 300 ppm for BIT, reducing the maximum admitted concentration, currently established at 360 ppm in Europe is recommended.

In their review, Silva et al. (2020) classified the four tested isothiazolinones by their respective levels of biocidal activity, from the highest to the lowest: MCI, OIT, BIT, and MI. This classification was paralleled with the cytotoxicity of these four compounds. However, direct comparison with our results is challenging. Our results indicated a clear sensitizing response when skin was exposed to MI at 30 ppm and OIT at 300 ppm, while exposure to BIT at 300 ppm suggested a potential sensitizing or irritation response. Conversely, skin exposure to MCI at 30 ppm and MI at 600 ppm appeared to induce more of an irritant than a sensitizing response. Consequently, MI acted as a skin sensitizer at 30 ppm, and OIT at 300 ppm. However, there was no clear classification of the sensitizing response of these compounds. Further testing of additional concentrations would be needed to determine threshold concentrations for sensitization and clarify the distinction between irritant and sensitizing responses, particularly for MI at 600 ppm compared to 30 ppm. Understanding these pathways is essential for recommending safe concentrations of isothiazolinone in products.

### Limitation of the study.

The study could not statistically validate the sensitization pathway for several isothiazolinones. This could be attributed to the use of unsuitable test doses, such as MCI dose, which may have been too low to observe a sensitization cascade. Alternatively, it is possible that some of the tested isothiazolinones, including MI at 600 ppm, MCI, and BIT, are skin irritants rather than skin sensitizers. Nevertheless, we demonstrated that the assessment procedure for potential sensitizing compounds could be optimized through the implementation of protocols for skin sample preparation and staining, as well as the integration of an automated method to assess DAB-stained pixel migration across the epidermis and penetration into the dermis.

The scripts are provided separately for two reasons. First, our developed pixel classifier is not a universal epidermis, dermis, squamous detector and will fail due to data drift when applied to new data. This detection part is thus, decoupled so, that multiple classifiers can be trained and tested. The second reason is that it provides a natural flow where the user can validate the accuracy, and correct, the classifications as needed before running the next script. The second script is much more robust, as long as the epidermis classification is as accurate as possible. While this approach can be considered time-consuming, it is much faster and reproducible than a fully manual approach of annotating the dermis and epidermis and localizing all brown DAB-positive areas. The proposed algorithm separates the annotated squares into two distinct non-touching regions and yields inaccurate results if this condition is not met. Considering the layered nature of skin and the fact we are working with transversal skin sections, we deemed this requirement is feasible.

In this study, we decided not to assess the LCs into the dermis because we are using an ex vivo skin model where there is no blood or lymph flow. Therefore, we believe that the absence of circulation can impact LC migration through the dermis*.* It is probable that the 24-h exposure to water was causing stress to the skin cells, which could result in the migration of Langerhans cells through the epidermis. In future studies, exposure duration to biocide solutions should be revised. To reduce the reaction caused by long exposure to water on the skin, exposure time could be decreased with usage of an insert instead of a patch. Alternatively, other vehicles such as solvents (e.g., ethanol (Na et al. 2021) or DMSO (Ouwehand et al. 2008)) could be used. The benefit of using solvents would be that they evaporate, leaving only the substance in contact with the epidermis. However, solvents can also be responsible for increased permeation of compounds through the skin (Berthet et al. 2017). In cosmetics, there is a high percentage of water, making it relevant to test sensitization with this vehicle. Based on the regulation threshold, this study focused on high concentrations for the BIT and OIT. It would be of interest to investigate any cutoff point where no Langerhans cells migration could be observed.

## Conclusion

In this study a human viable skin sensitization organo-culture model (SSM) was developed and validated to estimate the DAB-stained pixel migration across the epidermis in response to a skin exposure to four different biocides identified as sensitizers. For the analysis of the DAB-stained pixel distribution across the epidermis, we developed an open-source script to distinguish between three distinct skin tissue features: stratum corneum, epidermis, and dermis. The script provided graphical representation of the distribution with DAB-stained pixel through the epidermis using QuPath, a uniquely comprehensive, user-friendly, open-source bioimage analysis platform. The scripts, available at https://github.com/BIOP/qupath-skin-migration, may be used for the analysis of any type of cell staining in human skin. A link is also available to test the script on images.

Our results have shown that skin exposed to water in an occlusive setup significantly modified the Langerhans cells distribution across the epidermis suggesting that water could be considered as an irritant. Among the tested isothiazolinones, MI and BIT showed a different migration pattern than OIT-exposed skin. OIT-exposed skin presented a pattern where Langerhans cells were mostly located on the basal lamina as described in the literature for skin sensitizers, while MI and BIT showed a Langerhans cells irritant distribution.

Further steps could include the effect of MCI, MI, BIT, and OIT on primary keratinocytes to better understand the molecular pathway of sensitization.

## Supplementary Information

Below is the link to the electronic supplementary material.

Supplementary file1 Fig. S1 Distribution of DAB-stained pixel counts in the epidermis at different time points of each tested skin (n = 10). Control skin (no exposure) at the day of surgery. B: Control skin (no exposure) after 24 h of stabilization, when exposure started. C: Control skin (no exposure) after 24 h exposure (around 48 h after the surgery). Each violin plot represents the mean of sample replicates for each skin sample (PNG 244 KB)

Supplementary file2 (DOCX 15 KB)

## Data Availability

The authors confirm that the data supporting the findings of this study are available within the article and its supplementary materials.

## Abbreviations

BIT: Benzothiazolinone
CD1a: Cluster of differentiation 1a
DAB: 3,3′-Diaminobenzidine staining
MCI: Methylchloroisothiazolinone)
MI: Methylisothiazolinone
OIT: Octylisothiazolinone
