# Supplementary figures and images for: Development of image analysis tool to evaluate Langerhans cell migration after exposure to isothiazolinones
Source: Arch Toxicol. 2025 Mar 13;99(6):2463–77. doi: 10.1007/s00204-025-04013-3 (PMC12185578; doi:10.1007/s00204-025-04013-3)

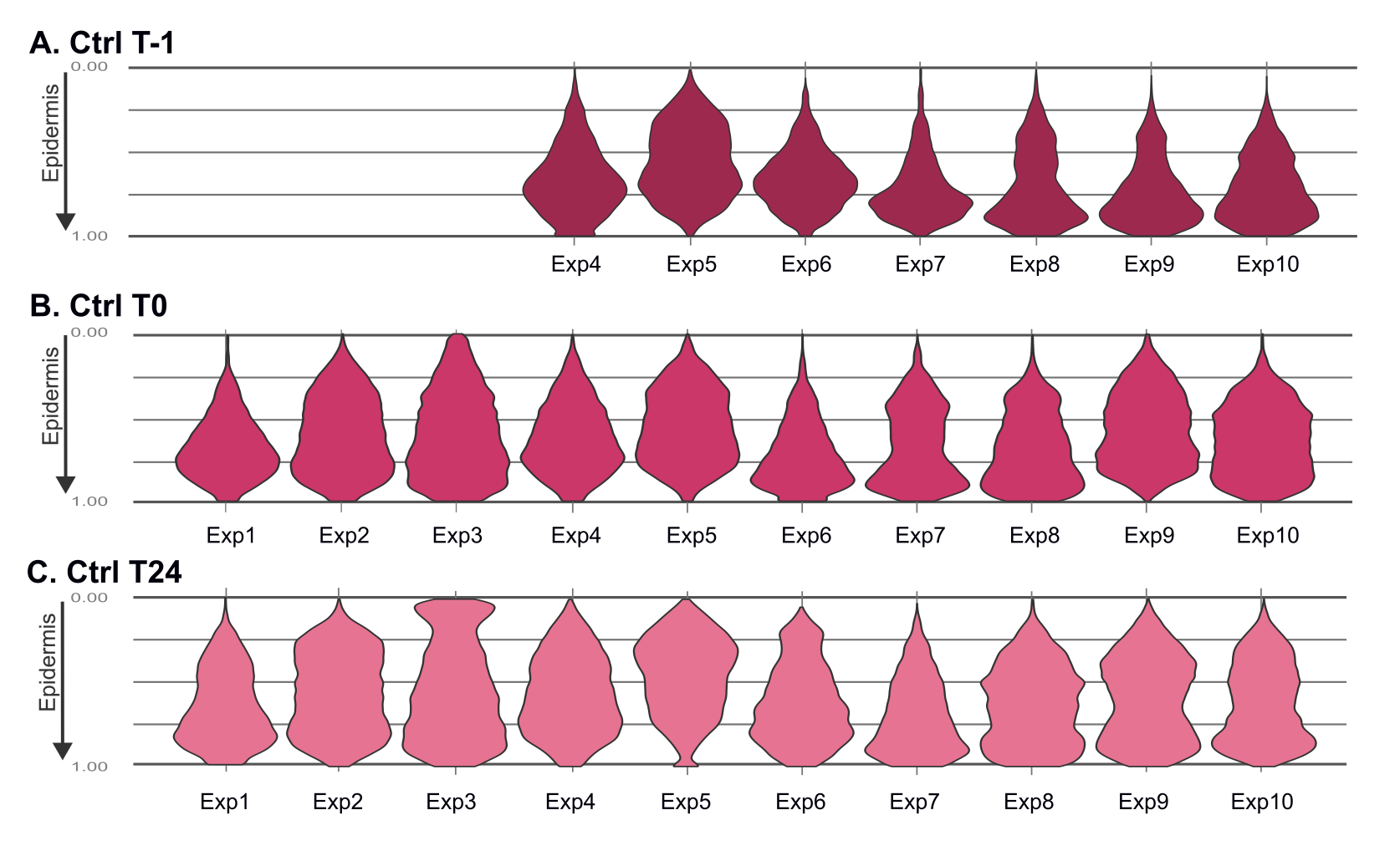

Supplement: Supplementary file 1 — Supplementary file1 Fig. S1 Distribution of DAB-stained pixel counts in the epidermis at different time points of each tested skin (n = 10). Control skin (no exposure) at the day of surgery. B: Control skin (no exposure) after 24 h of stabilization, when exposure started. C: Control skin (no exposure) after 24 h exposure (around 48 h after the surgery). Each violin plot represents the mean of sample replicates for each skin sample (PNG 244 KB) [file 204_2025_4013_MOESM1_ESM.png]
