# Supplementary material for: Development of image analysis tool to evaluate Langerhans cell migration after exposure to isothiazolinones
Source: Arch Toxicol. 2025 Mar 13;99(6):2463–77. doi: 10.1007/s00204-025-04013-3 (PMC12185578; doi:10.1007/s00204-025-04013-3)
Supplement: Supplementary file 2 — Supplementary file2 (DOCX 15 KB) [file 204_2025_4013_MOESM2_ESM.docx]

**Table S1.** Step-by-step experimental procedure

| Step | |
| --- | --- |
| 1 | Remove subcutaneous fat with scissors and scalpels |
| 2 | Clean the skin surface with Phosphate Buffered Saline 1x solution containing 1% antibiotics (penicillin/streptomycin) |
| 3 | Cut into squares put in a 12-well plate containing cell culture medium |
| 4 | Stabilize for 24 hours at 37°C, 95% humidity, and 5% CO2 |
| 5 | Placed the skin sample on an air-liquid bridge |
| 6 | Dry the skin surface |
| 7 | Place an insert (bottomless reservoir) on top of the skin |
| 8 | Fill into the exposure reservoir with test liquid (isothiazolinones (aq)) |
| 9 | Leave for exposure duration: 24 hours |
| 10 | Remove the exposure reservoir |
| 11 | Cut out exposed skin (round of 8 mm diameter) |
| 12 | Cut the sample in two (to have half circle) |
| 13 | Fix the sample in formalin for 24 hours at room temperature |
| 14 | Rinse the sample with PBS |
| 15 | Place the samples in 70% ethanol at 4C |
| 16 | Fix the samples in paraffin |
| 17 | Cut 7µm section tissue blocks included in paraffin at room temperature |
| 18 | Put sections on glass slides |
| 19 | Stain with immunohistochemistry (CD1a) |
| 20 | Scan the slide on microscope |
| 21 | Read the image on QuPath |
